# Research Trends and Hotspots Analysis Related to the Effects of Xenobiotics on Glucose Metabolism in Male Testes

**DOI:** 10.3390/ijerph15081590

**Published:** 2018-07-26

**Authors:** Yongsheng Fan, Guangxia Yu, Jun Yu, Jiantao Sun, Yu Wu, Xue Zhao, Yu Meng, Zhangdong He, Chunhong Wang

**Affiliations:** Department of Toxicology, School of Public Health, Wuhan University, DongHu Road 115, Wuhan 430071, China; fanyongsheng1993@163.com (Y.F.); yuguangxia@nbu.edu.cn (G.Y.); junyu6699@whu.edu.cn (J.Y.); 2015203050018@whu.edu.cn (J.S.); 2016203050018@whu.edu.cn (Y.W.); zhaoxuekk@sina.com (X.Z.); Mengyu9618@163.com (Y.M.); hzdadmin@126.com (Z.H.)

**Keywords:** xenobiotics, glucose metabolism, testes, bibliometrics, CiteSpace

## Abstract

This study aimed to integrate and analyze the existing studies and to explore research trends and hotspots related to the effects of xenobiotics on glucose metabolism in male testes. All articles were retrieved from the PubMed database, from an inception date up to 10 June 2017. CiteSpace software (version 5.1.R8 SE) was used for the co-word cluster analysis. A total of 165 eligible publications were included in this study. In 1949–1959, only two articles were published. After 1960, the number of articles increased steadily. These articles were published in 97 journals, in particular, in the *Indian Journal of Experimental Biology* (11 articles, 6.7%). Most of the authors (87.0%) only published one article. Only a few established research teams, mostly from the USA, worked consistently in this field. The main xenobiotics that had been studied were medicine and common environmental pollutants, e.g., gossypol, cadmium, di-n-butyl phthalate, and alpha-chlorohydrin. The hotspot keywords were Sertoli cell, lactate dehydrogenase, 6-phosphate dehydrogenase, oxidative stress, and glucose metabolism. The focus of research had been changed overtime. This is the first bibliometric study between xenobiotics and glucose metabolism in the male testes. The findings suggest that environmental pollutants have become a huge concern, and related research should be strengthened.

## 1. Introduction

Many agricultural and industrial chemicals introduced to the environment are deleterious to the development and reproductive systems of humans and animals [1]. Xenobiotics are chemicals foreign to the human system. They include a wide range of chemicals in our environment, e.g., persistent organic compounds, pesticides, heavy metals, organic solvents, medicine, and tobacco smoke. They are also defined as organic and inorganic compounds, which are produced by human beings and gradually introduced to the environment [2]. Continuous exposure to xenobiotics may have cumulative effects that lead to reproductive disorders [3]. For example, xenobiotics such as glyphosate are potentially toxic to sperm motility [4].

The testicles are a pair of organs that essentially perform two functions: sex steroid hormone biosynthesis and production of spermatozoa [5]. Glucose metabolism in the testes is critical for normal spermatogenesis and fertility [5,6]. Xenobiotics can affect the glucose metabolism at multiple levels, directly or indirectly, and impair spermatogenesis irreversibly [3,5,7].

Bibliometrics has been utilized to evaluate scientific output and the importance of scientific studies [8,9]. It is effective and useful in evaluating the scientific productions and research trends in a specific research field by word cluster analysis. Furthermore, it can identify the intellectual structures and research fronts by analyzing the most cited words.

In this study, a bibliometric analysis of xenobiotics on glucose metabolism in male testis research was carried out based on articles retrieved from the PubMed database, from an inception date to 10 June 2017. We presented publication trend by year, geographic regions, and most influential authors. We aimed to identify the intellectual structure, research trends, and hotspots of the effects of xenobiotics on glucose metabolism in male testes.

## 2. Methods and Materials

### 2.1. Search Strategy

PubMed database was searched from inception date up to 10 June 2017. Only studies in English were included. An auxiliary manual retrieval was performed to prevent missing studies. The keyword retrieval strategy was as follows: (1) nutrient or nutrition; (2) heavy metal; (3) organic pollutant or organic compound or organic chemical or organic solvent or benzene or cyanide or phenol; (4) pesticide; (5) terms (1) or (2) or (3) or (4); (6) testis or testes or testicle; (7) carbohydrate metabolism or glucose metabolism or metabolism of carbohydrates; and (8) terms (5) and (6) and (7).

### 2.2. Selected Criteria and Data Extraction

**Figure 1:**
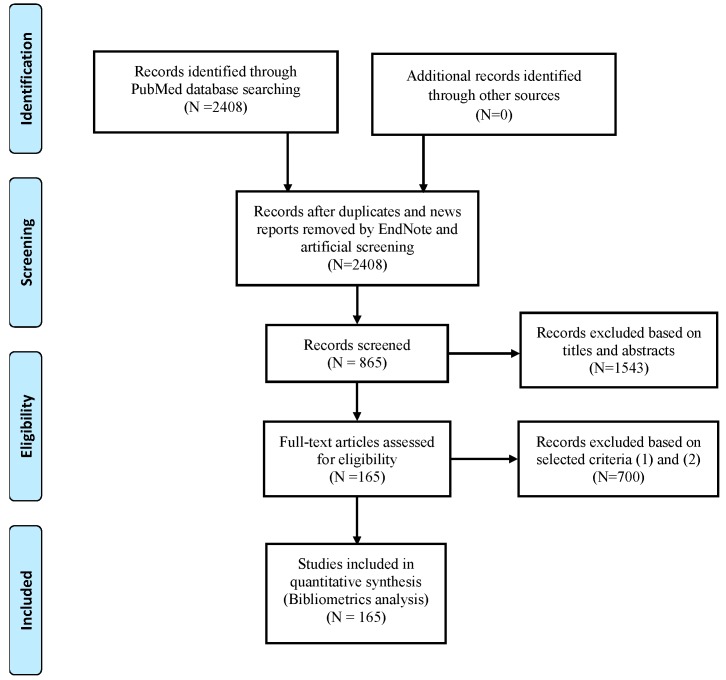
Flow diagram of study selection based on PRISMA 2009 guidelines [10] (PRISMA is an evidence-based minimum set of items for reporting in systematic reviews and meta-analyses).

Three authors (Jun Yu, Jiantao Sun, and Yu Wu) conducted the search independently. After removing the duplicates and news reports by EndNote X8, 2408 records were obtained. These data were extracted from the eligible studies according to the following criteria: (1) focused on male testis (including humans or animals, tissues or cells); (2) the contents of the study were about xenobiotics and glucose metabolism; and (3) written in English. The exclusion criteria included the following: (1) similar objective results in the same study or in the same institution at different time and (2) duplicates and news reports. Meanwhile, references were examined manually to identify any of the missing articles. If the full text of the included articles could not be obtained directly from the databases, we used the document delivery service from Wuhan University Library or directly contacted the author via email. The screening and review strategy is illustrated in Figure 1.

The collection of relevant data was extracted from the eligible studies. The main information included the title, year, corresponding author and his/her affiliation and country, journal title, xenobiotics, and experimental materials.

### 2.3. Analysis Methods

A descriptive analysis was used to present the characteristics of the included studies by publication years, countries, journals, and research teams.

To identify the intellectual structure and impacted works in the research field of xenobiotics on glucose metabolism in male testes, analyses were carried out in CiteSpace software (version 5.1.R8 SE). CiteSpace software (version 5.1.R8 SE) is a free Java-based application that was founded by Chaomei Chen (http://cluster.ischool.drexel.edu/~cchen/citespace/download/).

## 3. Results

As shown in Figure 1, 2408 publications were retrieved. After screening for article titles and abstracts, 865 publications remained. Finally, 165 eligible publications were included in this study based on these selected criteria formulated in advance (Table S1).

### 3.1. Characteristics of the Selected Studies

#### 3.1.1. Publication Years

**Figure 2:**
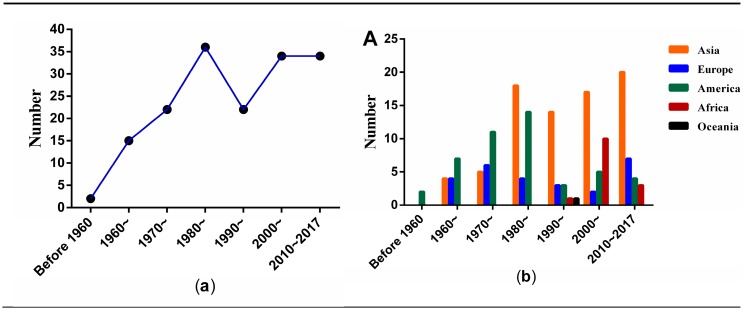
The distribution of 165 included articles: (**a**) by publication year; (**b**) by continents and year.

Figure 2a shows the number of publication of 165 articles by year. In 1949–1959, only two articles were published [10,11]. After 1960, the number of articles increased steadily from 15 in the 1960s to 34 in the 1980s. In the 1990s, the number declined to 22 papers and then increased to 34 in the first two decades of the new millennium.

According to the affiliation or country of the corresponding author, authors from five continents (Asia, America, Europe, Africa, and Oceania) contributed to this field (Figure 2b). Authors from Asia published approximately half of these papers (*N* = 78, 47.3%), followed by America (*N* = 46, 27.9%) and Europe (*N* = 26, 15.8%). American scholars were the first to start the research in this field. The first article was from the USA in 1949. However, after 1980, the number of articles from Asia exceeded the publications from America and Europe. Meanwhile, authors from Oceania only published one article in 1997 [12].

#### 3.1.2. Distribution of Articles by Countries, Journals, and Authors

The authors of the 165 articles were from 29 countries. As listed in Table 1, the top five countries are India, America, China, Egypt, and Portugal. Indian authors published approximately one third of these articles (*N* = 54, 32.7%). Authors in the USA published a total of 33 articles (20%) and ranked second. China, a developing country, had a steadily increasing article number in basic research and ranked third.

The 165 articles were published in 97 journals. Only five journals published no less than five articles. They were the *Indian Journal of Experimental Biology* (10 articles [13,14,15,16,17,18,19,20,21,22]), *Biology of Reproduction* (7 articles [23,24,25,26,27,28,29]), *Journal of Reproduction and Fertility* (6 articles [30,31,32,33,34,35]), *Endocrinology* (5 articles [10,36,37,38,39]), and *International Journal of Andrology* (5 articles [40,41,42,43,44]). A total of 26 journals (26.9%) published only 2–4 articles, and 66 journals (68.0%) published only one article. Therefore, no single journal is dominant over the other.

The 165 articles were published from 138 research teams. The top three productive authors for publications are shown in Table 2. The five authors published three articles or more. Satya P. Srivastava was the most productive author with five articles published [21,22,45,46,47]. The following three scientists were from the USA: Mannfred A. Hollinger [33,48,49,50] and Syed Husain [51,52,53,54], who published four articles, and Peter F. Hall [22,37,38], who published three articles. Pedro F. Oliveira from Portugal also published three articles [6,55,56]. For the remaining authors, the majority had only one article, indicating that only a few stable research teams were working in this field and mainly in the USA.

### 3.2. Main Research Topics

#### 3.2.1. Experimental Subjects and Xenobiotic Distribution

**Figure 3:**
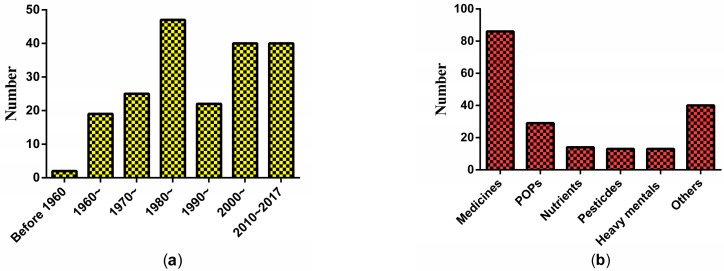
The distribution of xenobiotics studied related to glucose metabolism in testes: (**a**) by publication year; (**b**) types.

The 165 studies were conducted based on the population, animal models, and isolated tissues or cells. The animal models were the most used model to evaluate the association of xenobiotics on glucose metabolism in the testes, accounting for 84.8% of the articles. A total of 195 xenobiotics had been examined. Similar to the trend of publication years, the types of xenobiotics studied increased steadily (Figure 3a). All xenobiotics can be divided into six types: medicines, persistent organic pollutants (POPs), nutrients, heavy metals, pesticides, and others. Figure 3b illustrates that the most studied xenobiotics were medicines. Further analysis showed that several chemicals, e.g., gossypol, cadmium, di-*n*-butyl phthalate, alpha-chlorohydrin, cyclophosphamide, and delta-9-tetrahydrocannabinol, were frequently studied. Notably, cadmium, di-*n*-butyl phthalate, and alpha-chlorohydrin are all common environmental pollutants. Gossypol is a male contraceptive and also a residue in cottonseed oil. The findings implied that the effects of environmental pollutants on glucose metabolism in the testes are becoming a hotspot.

#### 3.2.2. Intellectual Structure and Hotspot Analysis

**Figure 4:**
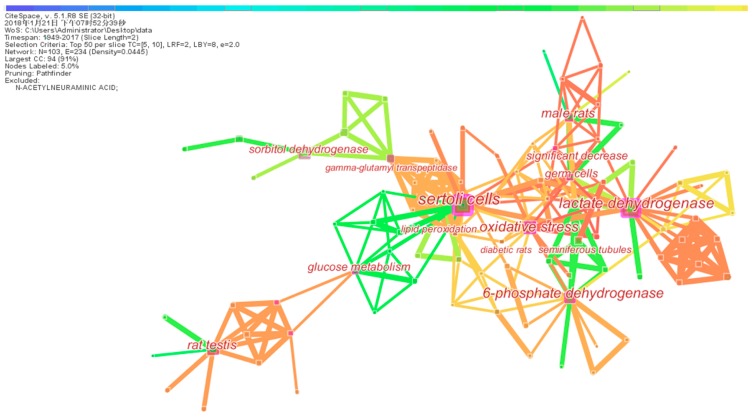
A network of co-words of 165 articles.

Network and cluster of co-words were applied to explore the intellectual structure and hotspot by CiteSpace software. From the map of co-words (Figure 4), 103 nodes and 263 connections were present between the keywords. The co-word clusters with more frequency and favorable silhouette presented large nodes. The top seven high-frequency clusters were Sertoli cell (SC), lactate dehydrogenase (LDH), 6-phosphate dehydrogenase, oxidative stress, rat testis, male rat, and glucose metabolism. The top seven favorable silhouette clusters were SC, LDH, glucose metabolism, 6-phosphate dehydrogenase, oxidative stress, gamma-glutamyl transpeptidase, and germ cell (Table 3). SC and LDH were the most used words by examining both the frequency and silhouette of co-word clustering. The other three hotspot keywords (6-phosphate dehydrogenase, oxidative stress, and glucose metabolism) were also significantly mentioned in these articles.

**Figure 5:**
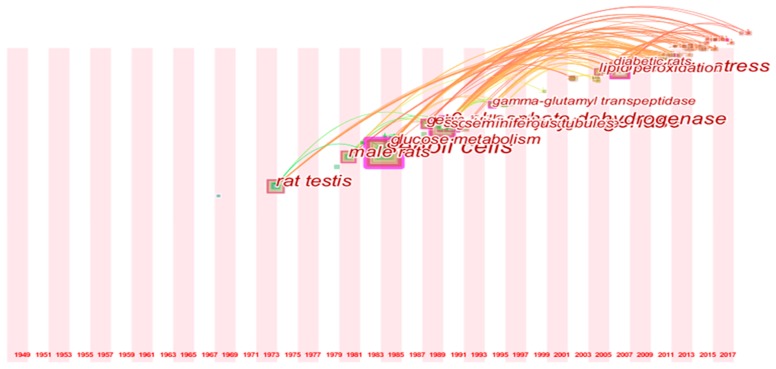
Timeline view for co-words analysis.

Timeline mapping of co-words was applied to further exhibit the research trends of this field. As illustrated in Figure 5, four noticeable turning points were observed in the term usage trend. Since 1972, “rat testis” was mostly used, as most of the studies were animal models. In the 1980s, the word “Sertoli cell” was introduced, as studies in isolated cells emerged in this field. In the late 1980s, more researchers began to study “lactate dehydrogenase” and “6-phosphate dehydrogenase” and this trend has changed into “oxidative stress” since 2006.

#### 3.2.3. Main Regulatory Pathways

**Figure 6:**
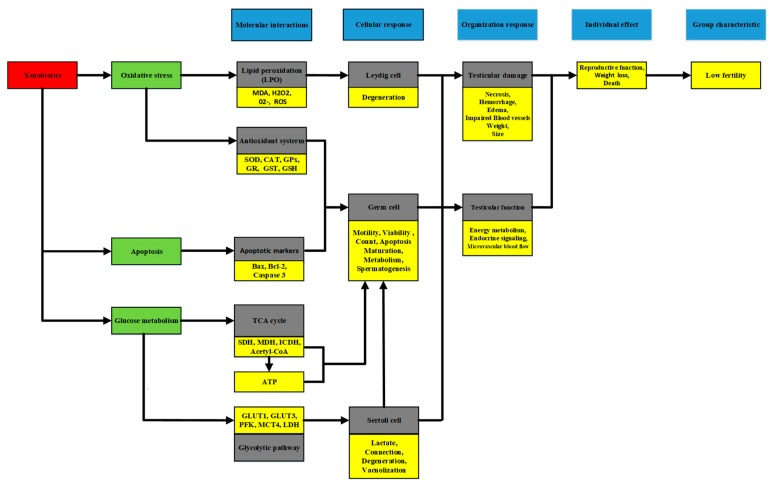
The main regulatory pathways induced by xenobiotics. Malondialdehyde (MDA), Superoxide radical (O_2_^−^), Hydrogen peroxide (H_2_O_2_), Reactive oxygen species (ROS), Peroxisome proliferator-activated receptor-gamma (PPAR-γ), Superoxide dismutase (SOD), Catalase (CAT), Glutathione peroxidase (GPx), Glutathione-S-transferase (GST), Glutathione reductase (GR), Reduced glutathione (GSH), Succinate dehydrogenase (SDH), Malate dehydrogenase (MDH), Isocitrate dehydrogenase (ICDH), Phosphofructokinase (PFK), Lactate dehydrogenase (LDH).

In accordance with previous studies, oxidative stress was a key factor in the etiology of male infertility, as demonstrated by enhanced lipid peroxidation and antioxidant defense system [57,58,59]. Another regulatory pathway, apoptosis, was presented by the balance of proapoptotic factor and antiapoptotic factors [1]. More details are provided in Figure 6.

In this study, the main changes induced by xenobiotics had more association with testicular glucose metabolism, and SCs metabolism was primarily affected [56,60]. Glycolysis was the main pathway for SCs to provide adequate energy substrate for themselves and germ cells. The expression of glucose transporter type 1 (GLUT1) and glucose transporter type 3 (GLUT3) was mainly distributed on the plasma membrane of SCs and was responsible for glucose transport [61]. For example, melatonin-exposed SCs presented higher GLUT1 expression [56], caffeine significantly increased the protein levels of GLUT1 and GLUT3 in human SCs [60], and both stimulated glucose uptake. Once glucose was transported into SC, the glycolytic pathway came into function, and phosphofructokinase was the rate-limiting control [62]. Glucose normally converted into lactate via LDH and transported across the plasma membrane to germ cells by specific monocarboxylate transporters (MCTs), particularly monocarboxylate transporter 1 (MCT1) and monocarboxylate transporter 4 (MCT4) [55]. The decline in their activities would lead to numerous abnormalities in SCs, including alterations in connection (the main component of the blood–testis barrier), cell degeneration, and vacuolization [63]. Thus, less lactate production and transportation was from SCs. Finally, it would cause defects in sperm maturation and spermatogenesis and even accelerated germ cell apoptosis. A study showed that testis exposed to xenobiotics have altered tricarboxylic acid (TCA) cycle due to testicular enzymes (SDH, Sorbitol dehydrogenase; MDH, Malate dehydrogenase; ICDH, Isocitrate dehydrogenase) in the mitochondrial fractions [59]. The pachytene spermatocyte maturation of the germinal epithelium was associated with SDH. The decreased SDH activity was mainly attributed to the reduced aerobic oxidation of acetyl CoA and ATP [64]. ATP served as an energy substance, and the loss of sperm motility was possibly correlated with low generation of ATP by xenobiotic-induced mitochondrial impairment [65].

Therefore, the balance loss in the main regulatory pathways would result in testicular damage and testicular dysfunction. The testicular damage included impaired vessels, changes in testicular size and weight with irreversible edema, hemorrhage, and necrosis. The testicular dysfunction induced by xenobiotics in energy metabolism, endocrine signaling, and microvascular blood flow would lead to body weight loss and reproductive problems, even death in individual effect. Finally, group characteristic presented low fertility.

## 4. Discussion

In this article, we presented the characteristics and research hotspot of the selected studies in the field of xenobiotic effects on glucose metabolism in male testes from 1949 to 2017. The first article was from the USA in 1949, but most published authors in this field were from Asia. Thus, developing countries, e.g., India and China, were more interested in researching this field.

Furthermore, the most studied xenobiotics were mainly medicine. The most frequently studied medicine was gossypol, a contraceptive causing infertility in humans and animals [66]. Gossypol was also a residue in cottonseed oil, and its side effects in male reproductive health should not be ignored [62,67]. Further analysis showed that cadmium, di-*n*-butyl phthalate, and alpha-chlorohydrin were in the top four chemicals studied, and these are common environmental pollutants. With the number of xenobiotics increasingly introduced to the environment, their impact on glucose metabolism dysfunction in male testes is becoming an important topic of research.

CiteSpace software was used to analyze research hotspots and emerging trends. Based on the co-word cluster analysis, hotspot keywords were Sertoli cell, lactate dehydrogenase, 6-phosphate dehydrogenase, oxidative stress, and glucose metabolism. The word with the highest frequency and the greatest silhouette was SCs. SCs are responsible for providing energy and nutritional support to germ cell development [5]. Germ cell development has specific metabolic requirements, preferentially using lactate as a substrate for ATP production. SCs produce lactate via the metabolism of various substrates, preferentially glucose. This is why SCs were the most valuable target for studying the deleterious effect of xenobiotics [1]. Meanwhile, some enzymes such as LDH and 6-phosphate dehydrogenase, which are associated with glucose metabolism, and ATP were also an active research area in recently published papers.

From timeline mapping of co-words, the focus of the research has changed over time. Before 1980, the effect of exogenous chemicals on testes was the key point; after 1980, the adverse effect of xenobiotics on glucose metabolism was studied in testicular cells [59,68]. In 1989, the research focus had shifted to enzymes related to testicular cell glucose metabolism, such as LDH and 6-phosphate dehydrogenase. Subsequently, the mechanism of how exogenous chemicals disrupted the glucose metabolism of testicular cells was also studied. Since 2006, one of the mechanisms, oxidative stress, has become a hotspot. Previous studies found that oxidative stress directly or indirectly interfered with enzyme activity and affected testicular carbohydrate metabolism [69,70,71].

Glucose metabolism was primarily affected by xenobiotics in the testes, including the process of TCA cycle and glycolytic pathway. The molecular interactions changed the specific markers during the process, resulting in alterations in the function and structure of Leydig cells, germ cells, and SCs to ameliorate the injury. Further testicular damage and dysfunction decreased fertility.

However, a few limitations must be considered for the bibliometric assessment. First, quantifying multiple literature problems is difficult. In particular, the literature system is very complex and unstable enough that we cannot obtain enough and effective information to reveal the macroscopic rule of the literature. Second, our articles were only from the PubMed databases. Third, bibliometrics depend on the support of mathematical and statistical techniques. Thus, some information may not have been analyzed.

## 5. Conclusions

To our knowledge, this study is the first bibliometric assessment of xenobiotics on glucose metabolism literature from 1949 to 2017. The number of published articles increased rapidly, especially in Asia. Medicines and environmental pollutants were the main xenobiotics studied. SCs were the most used models in these studies, and most studies focused on the glucose metabolism related to enzymes. Because environmental pollution is a huge concern and has a great impact on the human reproductive system, further research in this area will undoubtedly take place.

## Figures and Tables

**Table 1:** The 5 most productive countries for publications of effects of xenobiotics on glucose metabolism in testes.

Rank	Country	Continent	Articles	Percent (%)
1	India	Asia	54	32.7
2	USA	America	33	20.0
3	China	Asia	11	6.7
4	Egypt	Africa	8	4.9
5	Portugal	Europe	7	4.2

**Table 2:** The 5 most productive authors for publications of effects of xenobiotics on glucose metabolism in testes.

Author	Country	Publications	Percent (%)
Satya P. Srivastava	India	5	3.03
Mannfred A. Hollinger	USA	4	2.42
Syed Husain	USA	4	2.42
Peter F. Hall	USA	3	1.82
Pedro F. Oliveira	Portugal	3	1.82

**Table 3:** The top 7 co-word clusters with high frequency and silhouette in articles examining effects of xenobiotics on glucose metabolism in testes.

Frequency			Silhouette		
Rank	Frequency	Clusters	Rank	Silhouette	Clusters
1	25	Sertoli cell	1	0.84	Sertoli cell
2	17	lactate dehydrogenase	2	0.46	lactate dehydrogenase
3	14	6-phosphate dehydrogenase	3	0.37	glucose metabolism
4	13	oxidative stress	4	0.28	6-phosphate dehydrogenase
5	12	rat testis	5	0.24	oxidative stress
6	10	male rat	6	0.24	γ-glutamyl transpeptidase
7	8	glucose metabolism	7	0.20	germ cell

## Supplementary Materials

The following are available online at http://www.mdpi.com/1660-4601/15/8/1590/s1, Table S1: The list of 165 eligible publications.
