# Supplementary material for: Research Trends and Hotspots Analysis Related to the Effects of Xenobiotics on Glucose Metabolism in Male Testes
Source: Int J Environ Res Public Health. 2018 Jul 26;15(8):1590. doi: 10.3390/ijerph15081590 (PMC6121400; doi:10.3390/ijerph15081590)
Supplement: Supplementary file 1 [file ijerph-15-01590-s001.pdf]

Table S1. The list of 165 eligible publications.

Number	Title	Journal	Year	Author	Nation	Continent	Xenobiotics	Subjects
1	Protective effect of Satureja montana extract on cyclophosphamide-induced testicular injury in rats.	Chemico-biological interactions	2014	Nancy N.Shahin	Egypt.	Africa	cyclophosphamide	Male Wistar albino rats
2	Reversible anti-fertility effect of benzene extract of Ocimum sanctum leaves on sperm parameters and fructose content in rats.	Journal of basic and clinical physiology and pharmacology	2002	R. Nazeer Ahamed	India	Asia	a benzene extract of Ocimum sanctum leaves	albino rats
3	Melatonin and vitamin C exacerbate Cannabis sativa-induced testicular damage when administered separately but ameliorate it when combined in rats.	Journal of basic and clinical physiology and pharmacology	2016	Isiaka A. Alagbonsi	Nigeria	Africa	Cannabis sativa	albino rats
4	Metformin and male reproduction: effects on Sertoli cell metabolism.	British journal of pharmacology	2014	Marco G Alves	Portugal	Europe	Metformin	Wistar rats
5	Exposure to 2,4-dichlorophenoxyacetic acid alters glucose metabolism in immature rat Sertoli cells.	Reproductive toxicology (Elmsford, N.Y.)	2013	Marco G. Alves	Portugal	Europe	2,4-dichlorophenoxyacetic acid	Wistar rats
6	Mitochondrial dysfunction induced impairment of	European journal of pharmacology	2012	Hamdy A.A. Aly	Saudi Arabia	Asia	LPS	Wistar rats

	spermatogenesis in LPS-treated rats: modulatory role of lycopene.							
7	Modulatory role of lipoic acid on lipopolysaccharide-induced oxidative stress in adult rat Sertoli cells in vitro.	Chemico-biological interactions	2009	Hany A. El-Shemy	Egypt	Africa	LPS	albino rats
8	Bacterial lipopolysaccharide-induced oxidative stress in adult rat Sertoli cells in vitro.	Toxicology in vitro	2010	Hany A. El-Shemy	Egypt	Africa	Bacterial lipopolysaccharide (LPS)	albino rats
9	Potential testicular toxicity of sodium nitrate in adult rats.	Food and chemical toxicology	2010	Hamdy A. A. Aly	Egypt	Africa	sodium nitrate	albino rats
10	Effects of hyperglycemia on sperm and testicular cells of Goto-Kakizaki and streptozotocin-treated rat models for diabetes.	Theriogenology	2006	Joao Ramalho-Santos	Portugal	Europe	STZ	Wistar rats
11	THE ROLE OF GLUCOSE AND ACETATE IN THE OXIDATIVE METABOLISM OF THE TESTIS AND EPIDIDYMIS OF THE RAM.	The Biochemical journal	1963	Bye. F. Annison	Britain	Europe	glucose 、 acetate	Seventeen Merino rams
12	Chronic chromium exposure-induced changes in testicular histoarchitecture are associated with	Human reproduction (Oxford, England)	2005	M. Michael Aruldhas	India	Asia	chromium	monkeys

	oxidative stress: study in a non-human primate (Macaca radiata Geoffroy).							
13	Alpha-lipoic acid rebalances redox and immune-testicular milieu in septic rats.	Chemico-biological interactions	2011	Adel R. A. Abd-Allah	Saudi Arabia	Asia	LPS	albino rats
14	Histological and biochemical changes in testis of zinc deficient BALB/c strain of mice.	Indian journal of experimental biology	1994	Bedwal RS	India	Asia	zinc	BALB/c strain of mice.
15	Effects of gossypol on the antioxidant defense system of the rat testis.	Archives of andrology	1988	H.S. BENDER	USA	America	gossypol	Sprague-Dawley rats
16	Aqueous extract of pecan nut shell (Carya illinoensis [Wangenh.] K. Koch) exerts protection against oxidative damage induced by cyclophosphamide in rat testis.	Journal of environmental pathology, toxicology and oncology	2013	Dalila Moter Benvegnu	Brazil	America	Aqueous extract of pecan nut shell、cyclophosphamide	Wistar rats
17	Effects of plumbagin on reproductive function of male dog.	Indian journal of experimental biology	1984	SK. Bhargava	India	Asia	plumbagin	dogs
18	Toxic effects of phosphamidon on the testes of Swiss albino mice	Bulletin of environmental contamination and toxicology	1990	Pradeep Bhatnagar	India	Asia	Phosphamidon	albino mice
19	Influence of high tyrosine diet on the anterior pituitary and testis of the rat	Acta anatomica	1968	Narendra Mohan Biswas	India	Asia	high tyrosine diet	rats
20	[Activity of lactate dehydrogenase and	Voprosy pitaniia	1979	Bogatykh TA	Russia	Asia	chlorophos	Rats

	glucose-6-phosphate dehydrogenase activity in rat testicular tissues after exposure to certain pesticides].							
21	Role of glucose, fatty acids and protein in regulation of testicular growth and secretion of gonadotrophin, prolactin, somatotrophin and insulin in the mature ram.	Reproduction, fertility, and development	1997	Rachid Boukhliq	Australia	Oceania	glucose	Merino rams
22	Involvement of non-enzymatic antioxidant defenses in the protective effect of diphenyl diselenide on testicular damage induced by cadmium in mice.	Journal of trace elements in medicine and biology : organ of the Society for Minerals and Trace Elements (GMS)	2009	Cristina Wayne Nogueira	Brazil	America	diphenyl diselenide, cadmium	Swiss albino mice
23	Metabolic and testicular effects of the long-term administration of different high-fat diets in adult rats.	International braz j urol : official journal of the Brazilian Society of Urology	2015	Bianca Martins Gregório,	Brazil	America	high-fat diets	Wistar rats
24	Effects of carnitines on rat sertoli cell protein metabolism.	Hormone and metabolic research = Hormon- und Stoffwechselforschung = Hormones et metabolisme	2004	S. Palmero	Italy	Europe	carnitines	sertoli cell from Wistar rats
25	Iridoid glycoside from Cornus officinalis ameliorated diabetes mellitus-induced testicular damage in	Journal of ethnopharmacology	2016	Huiqin Xu	China	Asia	Streptozotocin, metformin, Iridoid glycoside	Wistar rats

	male rats: Involvement of suppression of the AGEs/RAGE/p38 MAPK signaling pathway.							
26	Effects of drugs on the reproductive physiology of male albino rats: Part II--Nicotine & morphine.	Indian journal of experimental biology	1978	N. J. Chinoy	India	Asia	Nicotine, morphine	Albino rats
27	Effects of drugs on the reproductive physiology of male albino rats: Part I--Central depressants & analgesic-antipyretics.	Indian journal of experimental biology	1978	N. J. Chinoy	India	Asia	ASA, metamizole	Albino rats
28	Bisphenol A impairs insulin signaling and glucose homeostasis and decreases steroidogenesis in rat testis: an in vivo and in silico study.	Food Chem Toxicol	2012	Premendu PrakashMathur	India	Asia	Bisphenol A	Wistar rats
29	Bisphenol A induces oxidative stress and decreases levels of insulin receptor substrate 2 and glucose transporter 8 in rat testi.	Reproductive sciences (Thousand Oaks, Calif.)	2012	Premendu PrakashMathur	India	Asia	Bisphenol A	rats
30	Treatment effect of l-Norvaline on the sexual performance of male rats with streptozotocin induced diabetes.	European journal of pharmacology	2016	Mamta F. Singh	India	Asia	streptozotocin/ l-Norvaline	albino rats
31	Roundup disrupts male reproductive	Free radical biology & medicine	2013	Ariane Zamoner	Brazil	America	calcium	Wistar rats

	functions by triggering calcium-mediated cell death in rat testis and Sertoli cells.							
32	Dose-dependent effects of caffeine in human Sertoli cells metabolism and oxidative profile: relevance for male fertility.	Toxicology	2015	Branca Maria Silva	Portugal	Europe	caffeine	people
33	[Enzyme histochemical and enzyme electrophoretic studies of rat testes in the first 48 hours of experimental cadmium intoxication].	Virchows Arch Pathol Anat Physiol Klin Med	1967	D. Knorre	Germany	Europe	cadmium	rat
34	Inhibition of lactate dehydrogenase C4 (LDH-C4) blocks capacitation of mouse sperm in vitro.	Cytogenetic and genome research	2003	Erwin Goldberg	USA	America	Oxamate	CD-1 mice
35	4-Nonylphenol induces disruption of spermatogenesis associated with oxidative stress-related apoptosis by targeting p53-Bcl-2/Bax-Fas/FasL signaling .	Environmental toxicology	2017	Kedi Yang	China	Asia	4-Nonylphenol	Sprague-Dawley (SD) rats
36	[Dynamic observation of carbofuran on symbolic enzymes in testis of rats].	Wei sheng yan jiu = Journal of hygiene research	2003	Duan Zhiwen	China	Asia	carbofuran	rats
37	Effects of alpha-chlorohydrin on the metabolism of	Journal of reproduction and fertility	1976	J.-L. Dacheux	France	Europe	alpha-chlorohydrin	rams

	testicular and epididymal spermatozoa of rams.							
38	Resveratrol alleviates diabetes-induced testicular dysfunction by inhibiting oxidative stress and c-Jun N-terminal kinase signaling in rats.	Toxicology and applied pharmacology	2015	Narayana Kilarkaje	Kuwait	Asia	Resveratrol	Wistar rats
39	Curcumin and kolaviron ameliorate di-n-butylphthalate-induced testicular damage in rats.	Basic & clinical pharmacology & toxicology	2007	Ebenezer Olatunde Farombi	Nigeria	Africa	di-n-butylphthalate(DBP)	Wistar rats
40	Tetracycline-induced reproductive toxicity in male rats: effects of vitamin C and N-acetylcysteine.	Experimental and toxicologic pathology	2008	Ebenezer Olatunde Farombi	Nigeria	Africa	Tetracycline	wistar rats
41	The insulin sensitiser metformin regulates chicken Sertoli and germ cell populations.	Reproduction (Cambridge, England)	2016	P. Froment	France	Europe	metformin	chickens
42	Comparison of the chromium distribution in organs and subcellular fractions of normal and diabetic rats by using enriched stable isotope Cr-50 tracer technique.	Biological trace element research	1999	Wenjun Ding	China	Asia	chromium	Wistar rats
43	The effect of carbon dioxide level and temperature on the in vitro metabolism of testis tissue.	Comparative biochemistry and physiology	1967	N. L. Vandemark	USA	America	carbon dioxide	rabbits

44	Energy metabolism of normal and lonidamine-treated Sertoli cells of rats.	Experimental and molecular pathology	1983	A. Floridi	Italy	Europe	lonidamine	rats
45	Effect of low doses of synthetic progestins on testicular function.	International journal of fertility	1972	K. Fotherby	Britain	Europe	synthetic progestins	fertile males
46	Metabolism of glucose-1-14C and glucose-6-14C by testis tissue from cryptorchid and testosterone propionate treated rabbits.	Endocrinology	1968	M. J. Free	USA	America	Testosterone Propionate	Testicular tissue slices from rabbits
47	Mechanism of testicular atrophy induced by di-n-butyl phthalate in rats. Part 1.	Journal of applied toxicology : JAT	1989	Masamichi Fukuoka	Japan	Asia	DBP(Di-n-butyl Phthalate)	Wistar rats
48	Mechanism of testicular atrophy induced by di-n-butyl phthalate in rats. Part 2. The effects on some testicular enzymes.	Journal of applied toxicology : JAT	1990	Yu Zhou	Japan	Asia	DBP(Di-n-butyl Phthalate)	Wistar rats
49	Participation of HIFs in the regulation of Sertoli cell lactate production.	Biochimie	2017	Silvina BeatrizMeroni	Argentina.	America	CoCl2	Sprague-Dawley rats
50	The AMP-activated protein kinase activator, 5-aminoimidazole-4-carboxamide-1-b-D-ribonucleoside, regulates lactate production in rat Sertoli cells.	Journal of molecular endocrinology	2007	Silvina Beatriz Meroni	Argentina	America	y 5-aminoimidazole-4-carboxamide-1-b-Dribonucleoside (AICAR)	Sprague–Dawley rats

51	Effect of glucose on ATP dephosphorylation in rat spermatids.	Journal of reproduction and fertility	1986	J. A. Grootegoed	Netherlands	Europe	lacate	rats
52	Effect of gossypol on cultured TM3 Leydig and TM4 Sertoli cells: 31P and 23Na NMR study.	NMR in biomedicine	1996	Jerzy W. Jaroszewski	Denmark	Europe	gossypol	Murine cells
53	Protective effect of ascorbic acid against ethanol-induced reproductive toxicity in male guinea pigs.	The British journal of nutrition	2013	M. Indira	India	Asia	ascorbic acid	guinea pig
54	Acute cadmium-induced changes in the energy metabolism of the rat testis.	Journal of reproduction and fertility	1970	M. Harkonen	Finland	Europe	cadmium	Sprague-Dawley rats
55	The male antifertility agents alpha chlorohydrin, 5-thio-D-glucose, and 6-chloro-6-deoxy-D-glucose interfere with sugar transport across the epithelium of the rat caput epididymidis.	Journal of andrology	1983	Barry T. Hinton	U.S.A	America	chlorohydrin, 5-thio-D-glucose, and 6-chloro-6-deoxy-D-glucose	epididymis
56	Effects of clomiphene on various biochemical reactions in the mouse testis in vitro.	Biochemical pharmacology	1971	Mannfred A. Hollinger	U.S.A.	America	Clomiphene	Swiss-Webster mice
57	Effect of nitrofurazone on the incorporation of L-lysine-U-14C into protein of rat testis.	Biochemical pharmacology	1966	Mannfred A. Hollinger	U.S.A.	America	Nitrofurazone	slices of testes
58	Effect of nitrofurazone on the aerobic	Journal of reproduction and fertility	1969	Mannfred A. Hollinger	U.S.A.	America	Nitrofurazone	Sprague-Dawley rats

	metabolism of uniformly labelled [14C]glucose in tissue slices of rat testes.							
59	Effect of puromycin and actinomycin D on glucose-stimulated protein and RNA labeling, in vitro, in rat testis.	Biochimica et biophysica acta	1972	Mannfred A. Hollinger	U.S.A.	America	puromycin and actinomycin D	Sprague-Dawley rats
60	Effect of in vivo and in vitro administration of clomiphene on RNA synthesis in rat testis.	Archives internationales de pharmacodynamie et de therapie	1972	M. A. Hollinger	USA	America	clomiphene	Sprague-Dawley rats
61	TiO2 Nanoparticle Exposure Decreases Spermatogenesis via Biochemical Dysfunctions in the Testis of Male Mice.	Journal of agricultural and food chemistry	2015	Fashui Hong	China	Asia	TiO_2 Nanoparticle	ICR (Imprinting Control Region) male mice,
62	[Bisphenol A alters glucose metabolism in rat Sertoli cells in vitro].	Zhonghua nan ke xue = National journal of andrology	2015	Huang W.	China	Asia	Bisphenol A	rat Sertoli cells
63	In vitro effect of adrenaline and other amines on glucose metabolism in sheep thyroid, heart, liver, kidney and testicular slices.	Biochemical pharmacology	1963	S. Hupka	Czechoslovakia	Europe	Adrenaline and other amines	Sprague-Dawley rats
64	Effects of delta-9-tetrahydrocannabinol on in vitro energy substrate metabolism in mouse and rat testis.	Physiology & behavior	1989	Syed Husain	U.S.A	America	delta-9-tetrahydrocannabinol	albino mice and Sprague-Dawley rats

65	Energy substrate metabolism in testis of rats treated with delta-9-tetrahydrocannabinol (THC) and cocaine (COC).	NIDA research monograph	1989	Syed Husain	U.S.A	America	delta-9-tetrahydrocannabinol (THC)	rats
66	Characteristics of cocaine interaction with delta-9-tetrahydrocannabinol on glucose metabolism in the rat testis.	Pharmacology, biochemistry, and behavior	1991	Syed Husain	U.S.A.	America	delta-9-tetrahydrocannabinol (THC)	Sprague Dawley rats
67	Rat testicular tissue glucose metabolism in the presence of delta-9-tetrahydrocannabinol.	Proceedings of the Western Pharmacology Society	1979	S . Husain	USA	America	delta-9-tetrahydrocannabinol (THC)	Sprague Dawley rats
68	Inhibitory effects of delta 9-tetrahydrocannabinol on glycolytic substrates in the rat testis	Pharmacology	1981	Syed Husain	U.S.A.	America	delta-9-tetrahydrocannabinol (THC)	Sprague Dawley rats
69	Altered biochemical responses by rat Sertoli cells and peritubular cells cultured under simulated diabetic conditions	Diabetologia	1984	J. C. Hutson	U.S.A	America	glucose, β-OHB, mannitol, sodium bicarbonate	Sertoli cells and peritubular cells
70	Effect of alpha-tocopherol supplementation on the impact of aflatoxin B1 on the testes of rats.	Experimental and toxicologic pathology	1998	Isaiah Nnanna Ibeh	Nigeria	Africa	aflatoxin Bl	albino rat
71	Biochemical changes in rat testicular cells in vitro produced by cannabinoids and	Toxicology and applied pharmacology	1977	Alexander Jakubovic	Canada	America	Cannabinoids, alcohol	Testicular tissue, Testicular Cell

	alcohol: metabolism and incorporation of labeled glucose, amino acids, and nucleic acid precursors.							
72	Pre- and postnatal toxicity of diazinon induces disruption of spermatogenetic cell line evidenced by increased testicular marker enzymes activities in rat offspring.	Journal of environmental pathology, toxicology and oncology	2013	Srinivasa Jayachandra	India	Asia	Diazinon	Sprague-Dawley rats
73	Effect of progesterone on biochemical composition of rat seminiferous tubules.	Indian journal of experimental biology	1970	Quaiser Jehan	India	Asia	progesterone	albino rats
74	Effects of ketoconazole (an imidazole antifugal agent) on the fertility and reproductive function of male mice.	Acta Europaea fertilitatis	1994	S. C. Joshi	India	Asia	ketoconazole	mice
75	Curcumin attenuates testicular damage, apoptotic germ cell death, and oxidative stress in streptozotocin-induced diabetic rats.	Molecular nutrition & food research	2013	Mehmet Kanter	Turkey	Asia	streptozotocin	Wistar albino male rats
76	Effect of N,N' -bis(dichloroacetyl)-1,8-octamethylenediamine on the chemical composition of the rat seminiferous tubules.	International journal of fertility	1966	Amiya B. Kar	India	Asia	N,N' -bis(dichloroacetyl)-1,8-octamethylenediamine	Rats
77	Effect of low doses of alpha chlorohydrin on	International journal of andrology	1981	S . Kaur	India	Asia	alpha chlorohydrin	Wistar rats

	the enzymes of glycolytic and phosphogluconate pathways in the rat testis and epididymis.							
78	1,2-dibromo-3-chloropropane (DBCP)-induced infertility in male rats mediated by a post-testicular effect.	Toxicology and applied pharmacology	1983	Charles River	USA	America	1,2-dibromo-3-chloropropane (DBCP)	rats
79	Protective role of pectin against Protective role of pectin against cadmium-induced testicular toxicity and oxidative stress in rats-induced testicular toxicity and oxidative stress in rats.	Toxicology mechanisms and methods	2013	Khaled M. M. Koriem	Malaysia.	Asia	cadmium	Wistar rats
80	Steroidogenic alterations in testes and sera of rats exposed to trichloroethylene (TCE) by inhalation.	Human & experimental toxicology	2000	KK Dutta	India	Asia	trichloroethylene (TCE)	Wistar rats
81	Mono-(2-ethylhexyl) phthalate targets glycogen debranching enzyme and affects glycogen metabolism in rat testis.	Toxicological sciences	2009	Hiroshi Handa	Japan	Asia	Mono-(2-ethylhexyl) phthalate	Rats
82	Interventional effects of squid ink polysaccharides on cyclophosphamide-	Bratislavske lekarske listy	2015	Liu HZ	China	Asia	cyclophosphamide	Kunming mice

	associated testicular damage in mice.							
83	Protein biosynthesis in the testis. II. Role of adenosine triphosphate (ATP) in stimulation by glucose.	Endocrinology	1968	Anthony R. Means	U.S.A	America	glucose	rats
84	Effects of chronic administration of Stevia rebaudiana on fertility in rats.	Journal of ethnopharmacology	1999	M. S. Melis	Brazil	America	Stevia rebaudiana	Wistar rats
85	Possible role of arachidonic acid in the regulation of lactate production in rat Sertoli cells.	International journal of andrology	2003	Selva B. Cigorraga	Argentina	America	arachidonic acid	Sertoli cells
86	Preliminary studies on the effects of cyproterone acetate on sexual activity and testicular function in adult male rhesus monkeys (Macaca mulatta).	Advances in the biosciences	1973	Richard P. Michiael	USA	America	cyproterone acetate	monkey
87	Histochemical studies of the rat epididymis after treatment with alpha-chlorohydrin (U-5897).	Folia histochemica et cytochemica	1974	K. Mietkiewski	Poland	Europe	alpha-chlorohydrin (U-5897)	rats
88	The effect of podophyllotoxin on tissue metabolism and enzyme systems.	The Journal of experimental medicine	1949	Zelma Baker Miller	USA	America	podophyllotoxin	Tissue Slice
89	Effect of aqueous leaf extract of Azadirachta indica on the reproductive organs in male mice.	Journal of Basic and Clinical Physiology and Pharmacology	2005	Musa Toyin Yakubu	Nigeria	Africa	aqueous leaf extract of Azadirachta indica	albino rats

90	Testicular toxicity of thiram in rat: morphological and biochemical evaluations.	Industrial health	1993	Vinod K. Mishra	India	Asia	Thiram	Albino rats
91	The influence of insulin and insulin-like growth factor-I on hexose transport by Sertoli cells.	Endocrinology	1985	Peter F. Hall	U.S.A	America	Insulin and Insulin-Like Growth Factor-I	Sertoli cells
92	Metabolism of round spermatids from rats: lactate as the preferred substrate.	Biology of reproduction	1982	Peter F. Hall	U.S.A	America	Pyruvate, Lactate	cells
93	Stimulation by follicle-stimulating hormone of synthesis of lactate by Sertoli cells from rat testis.	Endocrinology	1982	Peter F. Hall	U.S.A	America	FSH	Sertoli cells
94	Effects of hydro-alcoholic extract of Launaea acanthodes on serum gonadotropin and testosterone levels and the structure of seminiferous tubules in hyperglycemic rats.	Chinese journal of integrative medicine	2016	Morteza Behnam-Rassouli	Iran	Asia	STZ	albino Wistar rats
95	Testicular oxidative damage and role of combined antioxidant supplementation in experimental diabetic rats.	Journal of physiology and biochemistry	2011	Magda Mohasseb	Egypt	Africa	STZ	albino rats
96	Hexose phosphate levels in testes of galactose-fed rats.	Journal of reproduction and fertility	1971	G. I. Moonsammy	USA	America	galactose	Holtzman rats

97	Interleukin 1alpha stimulates lactate dehydrogenase A expression and lactate production in cultured porcine sertoli cells.	Biology of reproduction	1998	Mohamed Benahmed	France	Europe	Interleukin 1alpha (IL-1a)	Sertoli cells
98	Effect of insulin deprivation on metabolism and metabolism-associated gene transcript levels of in vitro cultured human Sertoli cells.	Biochimica et Biophysica Acta	2012	Pedro F. Oliveira	Portugal	Europe	insulin	Sertoli cells
99	Comparison of the effects of insulin and follitropin on glucose metabolism by Sertoli cells from immature rats.	Molecular and cellular endocrinology	1985	Ria B. Oonk	Netherlands	Europe	Insulin, FSH	Rat Sertoli cells
100	Differential effects of follicle-stimulating hormone, insulin, and insulin-like growth factor I on hexose uptake and lactate production by rat Sertoli cells.	Journal of cellular physiology	1989	Ruud Jansen	U.S.A	America	Insulin, FSH, IGF-I	Rat Sertoli cells
101	3-(4-hydroxy phenyl)-2-propenoic acid - a reproductive inhibitor in male rat.	Contraception	1981	Anita Pakrashi	India	Asia	3-(4-hydroxy phenyl)-2-propenoic acid	rats
102	Metabolic effects of L-carnitine on prepubertal rat Sertoli cells.	Hormone and metabolic research	2000	S. Palmero	Italy	Europe	L-carnitine	Sertoli cell
103	Effect of lead and cadmium co-exposure on testicular steroid	Andrologia	2012	Sarita Gupta	India	Asia	lead 、 cadmium	virgin Charles Foster rats

	metabolism and antioxidant system of adult male rats.							
104	Spermatotoxic effects of Cananga odorata (Lam): a comparison with gossypol.	Fertility and sterility	2009	Indira Madambath	India	Asia	Cananga odorata (Lam), gossypol	albino rats
105	Effect of oral administration of carbofuran on male reproductive system of rat.	Human & experimental toxicology	1995	Satya P. Srivastava	India	Asia	carbofuran	albino rats
106	Effects of carbaryl on the rat's male reproductive system.	Veterinary and human toxicology	1995	Pant, N.	India	Asia	carbaryl	Rats
107	Carbohydrate metabolism studies on the testis of rats fed certain nitrofurans.	Endocrinology	1953	Henry E. Paul	USA	America	Furacin	albino rats
108	Effect of alpha-chlorohydrin on metabolism and testosterone secretion by rat testicular interstitial cells.	International journal of andrology	1985	Gedalia Paz	Israel	Asia	alpha-chlorohydrin	Interstitial cells
109	Effect of the antifertility agent, gossypol acetic acid, on the metabolism and testosterone secretion of isolated rat interstitial cells in vitro.	Contraception	1984	G. F. Paz	Israel	Asia	gossypol	cells
110	Effect of dermal application of hexachlorocyclohexane (HCH) on male	Human & experimental toxicology	1995	Satya P. Srivastava	India	Asia	1,2,3,4,5,6-hexachlorocyclohexane (HCH)	Wistar rats

	reproductive system of rat							
111	Modulation of antioxidant defense system by the environmental fungicide carbendazim in Leydig cells of rats.	Reproductive toxicology (Elmsford, N.Y.)	2007	Bassouvalingam Kumaran	India	Asia	fungicide carbendazim	albino rats
112	myo-Inositol metabolism in rat testis in response to streptozotocin-induced diabetes.	Archives of biochemistry and biophysics	1980	William W. Wells	U.S.A	America	streptozotocin	rats
113	Effect of gossypol on the fertility of male rats.	Acta Europaea fertilitatis	1990	N. R. Kalla	India.	Asia	gossypol	Rats
114	Effect of prolactin inhibition by bromocriptine on testicular metabolism in the adult rat.	International journal of andrology	1982	M. Ramachandra Rao	India	Asia	bromocriptine	Wistar strain albino rats
115	Curcumin ameliorates testicular damage in diabetic rats by suppressing cellular stress-mediated mitochondria and endoplasmic reticulum-dependent apoptotic death.	Biochimica et biophysica acta	2015	Parames C. Sil	India	Asia	streptozotocin	Wistar rats
116	Metabolic modulation induced by oestradiol and DHT in immature rat Sertoli cells cultured in vitro.	Bioscience reports	2012	Pedro F. Oliveira	Portugal	Europe	E2 (17β-oestradiol), DHT (dihydrotestosterone)	Sertoli cell
117	Effect of 2-bromo-alpha-ergocryptine (CB 154) on plasma	Biology of reproduction	1977	J. P. Ravault	France	Europe	2-bromo-alpha-ergocryptine (CB 154)	Ile-de-France lambs

	prolactin, LH and testosterone levels, accessory reproductive glands and spermatogenesis in lambs during puberty.							
118	Specificity of gossypol uncoupling: a comparative study of liver and spermatogenic cells.	The American journal of physiology	1988	Juan Reyes	Chile	America	gossypol	Spermatogenic cells
119	A bioenergetic model of gossypol action: effects of gossypol on adult rat spermatogenic cells.	The American journal of physiology	1988	Juan Reyes	Chile	America	Gossypol, carbonyl cyanide m-chlorophenylhydrazone (CCCP)	spermatogenic cell
120	Energy metabolism of cultured TM4 cells and the action of gossypol.	Biology of reproduction	1986	Dale J. Benos	Britain	Europe	gossypol	TM_4 cells
121	Melatonin alters the glycolytic profile of Sertoli cells: implications for male fertility.	Molecular human reproduction	2014	Pedro F. Oliveira	Portugal.	Europe	Melatonin	Sertoli cell
122	Effects of diallyl sulfide and zinc on testicular steroidogenesis in cadmium-treated male rats.	Journal of biochemical and molecular toxicology	2008	Nermin A. H. Sadik	Egypt	Africa	cadmium	albino rats
123	Effect of isoproturon on male reproductive system: clinical, histological and histoenzyonological studies in rats.	Indian journal of experimental biology	1997	S. N. Sarkar	India	Asia	isoproturon	Rats
124	Lead induced testicular changes in	Folia histochemica et cytobiologica	1989	S. V. Chandra	India	Asia	Lead	Rats

	protein malnourished rats.							
125	Protective effect of DL-alpha-lipoic acid in cyclophosphamide induced oxidative injury in rat testis.	Reproductive toxicology (Elmsford, N.Y.)	2004	Palaninathan Varalakshmi	India	Asia	cyclophosphamide	albino rats
126	Beneficial effects of DL-alpha-lipoic acid on cyclophosphamide-induced oxidative stress in mitochondrial fractions of rat testis.	Chemico-biological interactions	2005	PalaninathanVaralakshmi	India	Asia	cyclophosphamide	albino rats
127	Fertility control in vas occluded rats and the biochemical effects of ascorbic acid feeding.	Experimental and clinical endocrinology	1983	V. P. DIxIT	India	Asia	ascorbic acid	albino rats
128	Selected testicular enzymes as biochemical markers for procarbazine-induced testicular toxicity.	Archives of toxicology	1984	I. P. Lee	U.S.A	America	procarbazine	CD-1 male mice
129	Occurrence of oxidative impairments, response of antioxidant defences and associated biochemical perturbations in male reproductive milieu in the Streptozotocin-diabetic rat.	International journal of andrology	2007	Muralidhara	India	Asia	streptozotocin	rats
130	Chelation therapy and vanadium: effect on reproductive organs in rats.	Indian journal of experimental biology	2007	Sangeeta Shukla	India	Asia	vanadium	albino rats

131	Levels of sulfhydryls and sulfhydryl-containing enzymes in brain, liver and testis of manganese treated rats.	Archives of toxicology	1977	Satya V. Chandra	India	Asia	sulfhydryls	albino rats
132	Methallibure inhibition of testicular and seminal vesicle activity in catfish, Clarias batrachus (Linn.): a study correlating changes in serum sex steroid profiles.	Acta biologica Hungarica	2000	K. P. Joy	India.	Asia	Methallibure	Catfishes
133	Effect of protein malnutrition on sex organs of metanil yellow exposed male rats	Biomedical and environmental sciences	1998	R. L. Sing	India	Asia	metanil yellow	albino rat
134	Effect of nitrofurazone on the reproductive organs in adult male mice.	Asian journal of andrology	2001	Shio Kumar Singh	India	Asia	nitrofurazone	P mice
135	Aspects of the biochemical toxicology of cadmium.	Federation proceedings	1976	R. L. Singhal	Canada	America	cadmium	rats
136	Endosulfan-induced biochemical changes in the testis of rats	Veterinary and human toxicology	1995	N. Sinha	India	Asia	Endosulfan	Druckrey rats
137	Post-testicular antifertility effects of Abrus precatorius seed extract in albino rats.	Journal of ethnopharmacology	1990	Bakesh Sinha	India	Asia	Abrus precatorius seed extract	Rats
138	Effect of steroidal fraction of seeds of Abrus precatorius Linn. on rat testis.	Indian journal of experimental biology	1990	Seema Sinha nee Kulshreshtha	India	Asia	steroidal fraction of seeds of Abrus precatorius Linn.	Rats

139	Effects of ethanol treatment on Leydig cellular NADPH-generating enzymes and lipid profiles.	Endocrine journal	1995	Karundevi Balasubramanian	India	Asia	ethanol	Albino rats
140	Effect of TSAA-291 on male reproductive tract and fertility of the rat.	International journal of fertility	1989	A. Srivastava	India	Asia	TSAA-291	rats
141	Ninety-day toxicity and one-generation reproduction study in rats exposed to allethrin-based liquid mosquito repellent.	The Journal of toxicological sciences	2006	Rajendra Behari Raizada	India	Asia	liquid mosquito repellent (LMR)	Albino rats
142	Effect of styrene on testicular enzymes of growing rat.	Indian journal of experimental biology	1992	Satya P. Srivastava	India	Asia	styrene	albino rats
143	Testicular toxicity of di-n-butyl phthalate in adult rats: effect on marker enzymes of spermatogenesis.	Indian journal of experimental biology	1990	Satya P. Srivastava	India	Asia	di-n-butyl phthalate (DBP)	rats
144	Testicular effects of di-n-butyl phthalate (DBP): biochemical and histopathological alterations.	Archives of toxicology	1990	Satya P. Srivastava	India	Asia	di-n-butyl phthalate (DBP)	albino rats
145	Ascaridia galli: lactic acid production, glycogen content, glycolytic enzymes and properties of purified aldolase, enolase and glucose-6-phosphate dehydrogenase.	Parasitology	1970	C. R. Krishna Murti	India	Asia	Oxygen	Ascaridia gall

146	Assessment of reproductive toxicity in male rats following acute and sub-chronic exposures to diphenyl diselenide and diphenyl ditelluride.	Food and Chemical Toxicology	2006	Cristina Wayne Nogueira	Brazil	America	diphenyl diselenide and diphenyl ditelluride	Wistar rats
147	Effects of galactose on levels of free myo-inositol in rat tissues.	Annals of the New York Academy of Sciences	1969	Mark A. Stewart	U.S.A	America	galactose	rats
148	Energy metabolism of spermatozoa. IV. Effect of calcium on respiration of mature epididymal sperm of the rabbit.	Biology of reproduction	1975	Bayard T. Storey	U.S.A	America	calcium	White New Zealand rabbits
149	Effects of fluoroacetate on the testis of the rat	Journal of reproduction and fertility	1979	Judith L. Sullivan	USA	Asia	fluoroacetate	Male Sprague-Dawley rats
150	Repeated exposure to iron oxide nanoparticles causes testicular toxicity in mice.	Environmental toxicology	2017	Ekambaram Perumal	India	Asia	Fe_2O_3 -NPs	Albino mice
151	Effect of exogenous selenium on the testicular toxicity induced by ethanol in rats.	Indian journal of physiology and pharmacology	2006	M. Indira	India	Asia	ethanol/selenium	Albino rats
152	[Modifications of the accessory glands of male genital tract of intact and castrated rats after sulpiride treatment (author's transl)].	Annales d'endocrinologie	1979	Tahiri-Zagret C	France	Europe	Sulpiride	rats
153	Effects of quercetin on rat testis aerobic glycolysis.	Canadian journal of physiology and pharmacology	1995	Raquel Trejo	Mexico	America	quercetin	Dawley rats

154	The effects of steroid and gonadotrophic hormones in vitro on the metabolic activity of normal and cryptorchid rat testicular tissues.	Biology of reproduction	1970	W. R. Gomes	U.S.A	America	Steroid, gonadotrophic hormones	Wistar rats
155	Vitamin E ameliorates aflatoxin-induced biochemical changes in the testis of mice.	Asian journal of andrology	2001	Anita Nair	India	Asia	aflatoxin	Albino mice
156	Alpha-chlorohydrin-induced changes in the distribution of free myo-inositol and prostaglandin F2alpha, and synthesis of phosphatidylinositol in the rat epididymis.	Biology of reproduction	1974	J. K. Voglmayr	U.S.A	America	Alpha-chlorohydrin	Sprague-Dawley rats
157	Early events in rat testis after gossypol administration.	Acta Europaea fertilitatis	1990	N.R. Kalla	India	Asia	gossypol	albino rats
158	[Effects of ganoderma lucidum spores on cytochrome C and mitochondrial calcium in the testis of NIDDM rats].	Zhonghua nan ke xue = National journal of andrology	2006	Wang Shu—qiu	China	Asia	ganoderma	Wistar rats
159	[Intervention effect of ganoderma lucidum spores on the changes of XOD, MPO and SDH in the testis tissue of NIDDM rats].	Zhonghua nan ke xue = National journal of andrology	2008	Wang Shu— qiu	China	Asia	ganoderma	Wistar rats
160	Enzymes of myo-inositol and inositol lipid metabolism in rats with	The Biochemical journal	1979	John N. Hawthorne	Britain	Europe	Streptozotocin	Wistar rats

	streptozotocin-induced diabetes.							
161	Effects of oral administration of aqueous extract of Fadogia agrestis (Schweinf. Ex Hiern) stem on some testicular function indices of male rats.	Journal of ethnopharmacology	2008	Musa Toyin Yakubu	Nigeria	Africa	aqueous extract of Fadogia agrestis (Schweinf. Ex Hiern) stem	albino rats
162	Aluminium-induced changes in hemato-biochemical parameters, lipid peroxidation and enzyme activities of male rabbits: protective role of ascorbic acid.	Toxicology	2004	Mokhtar I. Yousef	Egypt.	Africa	aluminium chloride (AlCl3)/ascorbic acid (AA)	rabbits
163	Protective role of isoflavones against the toxic effect of cypermethrin on semen quality and testosterone levels of rabbits.	Journal of environmental science and health. Part. B, Pesticides, food contaminants, and agricultural wastes	2003	Mokhtar I. Yousef	Egypt	Africa	cypermethrin/isoflavones	rabbits
164	Strontium fructose 1,6-diphosphate alleviates early diabetic testopathy by suppressing abnormal testicular matrix metalloproteinase system in streptozocin-treated rats.	The Journal of pharmacy and pharmacology	2009	Han-Jie Ying	China	Asia	streptozocin/Strontium fructose 1,6-diphosphate	Sprague-Dawley rats
165	Zinc deficiency exacerbates diabetic down-regulation of	The Journal of nutritional biochemistry	2012	Wei Li	China	Asia	Zinc	FVB mice

	Akt expression and function in the testis: essential roles of PTEN, PTP1B and TRB3.
